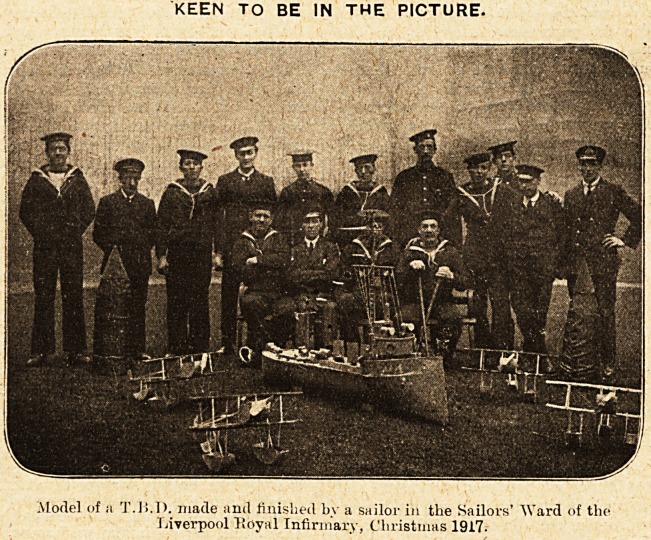# Bolo Buried and Forgotten Everywhere

**Published:** 1918-01-12

**Authors:** 


### OUR HOSPITALS' CHRISTMAS, 1917.

### BOLO BURIED AND FORGOTTEN EVERYWHERE.

### Welsh Hospital, Netley.

Christmas week has been kept at the Welsh Hospital, Netley, on lines very similar to those which were observed last year. On Christmas Eve the wards were visited by carol singers, who ended their tuneful pilgrimage at the Commanding Officer's quarters. On Christmas Day, thanks to the generous provision of an ample fund by kind friends in Wales, all the patients, 260 in number, partook of a dinner of turkey and plum-pudding, which was followed in the afternoon and evening by games and ward concerts. The medical and nursing staffs, according to the established custom of the hospital, had dinner together, during which they were visited by groups of orderlies and patients dressed in fancy costumes, who chanted verses of home manufacture which were full of topical and personal allusions. The rest of the week was filled by various ward entertainments and concerts. On Wednesday evening a concert was given by the orderlies to the rest of the staff and the officer patients, much amusement being caused by the verses sung by the Welsh Hospital choir in which everyone connected with the hospital was humorously and skilfully "taken off." In B Ward, which possessed among its patients an efficient stage manager and playwright, a sketch entitled "Black Justice" was given on Thursday, which was thoroughly enjoyed. Christmas-trees were to be found in two of the wards, the presents being distributed by Father Christmas to patients and to members of the staff. A very clever and sprightly Pierrot party, organised by Miss Turner, had a rousing reception in the two wards in which they gave their show on Friday evening, and the week's entertainments closed on Saturday with a tea given by the officer patients in E Ward to their comrades from the other wards. Over 200 patients were able to attend, many who were unable to walk being carried in and laid on the beds which had been placed lengthwise along the sides of the wards. An excellent concert concluded a most enjoyable afternoon. The ward decorations were as tasteful and varied as ever. Green branches traced the outlines of the cross-beams in a vista of arches, the green being relieved by small coloured paper flowers attached in hundreds to the twigs, the results being most effective and well worth the time and labour expended.

### Royal Victoria Infirmary, Newcastle-upon-Tyne.

Yuletide festivities at Newcastle Infirmary began on Christmas Eve, when the nursing staff promenaded the wards and corridors and sang carols to the patients, among whom are 200 soldiers. Immediately after breakfast, one of the house surgeons, in the garb of Father Christmas, visited each prettily decorated ward, and distributed presents to all the patients, the wounded soldiers receiving extra gifts of writing-pads, kindly given by Mr. Cooper, and five packets of cigarettes, presented by the Newcastle Chamber of Commerce. The Christmas service followed, the chapel, beautified by flowers and greenery, being crowded. The sermon was preached by the Senior Chaplain to the Forces in Newcastle (the Rev. Mr. Purvis), and the lessons were read by Sir George Hare Philipson. The usual Christmas fare was provided for dinner, and amongst those who helped to carve the turkeys and serve the plum-puddings were the chairman of the governors (Sir George Hare Philipson), Sir Riley Lord, General and Mrs. Montgomery, Colonel Angus, Colonel Richardson, and Mr. Charles Irwin. In the afternoon there were Christmas-trees for the children, and the little patients received their gifts at the hands of Santa Claus; while at night two excellent entertainments were given in every ward. Concerts were provided in all the wards during the afternoon and evening of Christmas Day, members of the entertainments committee having arranged the programme.

☛ The previous article appeared on January 5, 1918, p. 301.

### OUR HOSPITALS' CHRISTMAS, 1917.—(*continued*).

### Liverpool Royal Infirmary.

AFTER completing the decorations and other preparations, Christmas was ushered in on Christmas Eve after the lights were down by the nurses singing carols round every ward. They carried coloured lanterns, each different, designed and made by one of the soldiers. The whole effect gave much pleasure to all the patients, judging from the remarks the next day. Christmas Day began with celebration of Holy Communion at 6 A.M., when over one hundred members of the nursing and domestic staffs communicated. A second celebration, with service, was held at 10.30 A.M., when patients who were able attended, and the chaplain celebrated in each ward for the patients in bed. No turkeys were bought, but quite unexpectedly sufficient were sent by different friends for the naval and military wards, the other patients having joints of roast beef. The puddings were voted excellent by all, though lacking the usual ingredients; while there was an abundance of fruit, etc. The ward teas, from 3 P.M. onwards, were much enjoyed, even though the cakes lacked the usual "plumminess" and icings; the sisters always give special thought to the dinner and tea tables. At 4.30 the concerts began, when a troupe of nurses and residents went from ward to ward giving a capital programme of songs, choruses, and recitations. Other residents—of both sexes—gave a topical rendering of "Trial by Jury." These troupes were followed by many outside friends, nurses who were free, and the servants, making the whole atmosphere happy and friendly.

**KEEN TO BE IN THE PICTURE.**

Model of a T.B.D. made and finished by a sailor in the Sailors' Ward of the Liverpool Royal Infirmary, Christmas 1917.

THE DECORATIONS.

The civilian ward sisters have always concentrated on different designs in electric-light shades, and this year the whole effect was both prettier and more original than usual. The entrance hall and main corridor were softly glowing under pink shades, showing pen-and-ink sketches of "Eve" of the *Tatler*, billowing in the approved fashion of that lady. The soldiers' wards indulged in a very realistic snowstorm, numerous black cats of aggressive or placid mien, flags, and strings of pennons, with a model Tank made by the men, and lighted up. The sailors' ward was very typical, with no frivolous shades of many hues, but each light enclosed in a "shell"; one side of the ward was red and the other green, while the centre lights were "seaplanes." The centre of attraction, which is shown in our illustration, was a model of a destroyer, most complete in every detail, even to the tiny electric light over the "crow's nest," the guns being made to swivel in the most correct way. This model was over five feet in length, beautifully made and finished by a sailor, to whom it was a labour of love for some weeks.

The Christmas Fund again provided a suitable present for every patient. Each sailor and soldier had either a pigskin cigarette-case filled, and a cigar, or a pocket-case with pipe and tobacco for those who preferred it. These gifts were hung on four fine trees, which Lord Derby, as usual, most kindly presented to the naval and military wards. Christmas did, perhaps, lack the noise and jollity of pre-war years, but the general feeling was one of quiet happiness.

### Ancoats Hospital, Manchester.

A CHRISTMAS Fund was started early in December, which was so successful that each ward sister was given £6 wherewith to purchase decorations for the wards and extras for the patients. The matron, Miss Maud Earl, and her staff are to be congratulated upon the complete success of the Christmas festivities.

Sister Armstrong, in the soldiers' wards, had covered her electric lights with shades to represent old-fashioned lanterns. Through the kindness of friends a Christmas-tree was provided for each of her (two) wards, filled with presents for the wounded "Tommies." Mrs. Westwell most generously made a present of a £5 War Bond to each of the thirty-nine soldiers, which was much appreciated by the men, and which she presented to each one personally on Christmas morning. Sister Rogers, in charge of the women's wards, and Sister Owen, in charge of the male wards, tastefully decorated their wards with paper festoons and presented each of their patients with useful gifts.

To the children's wards, the pet wards of the hospital, especially at this season of the year, Sister Stephenson gave much time and personal service. A large Christmas-tree was provided by Messrs. Thelwell, of Manchester, which was lighted with coloured electric lamps and loaded with toys. The wards were decorated and electric lamps shaded with tinted paper, which gave a pretty effect, looking warm and cosy against the red-tiled walls and the white enamelled cots. The Christmas dinner to the soldiers was provided by the Lloyds Bank Wounded Soldiers Fund per Messrs. Rains and Miller, hon. secretaries, and consisted of turkey, Christmas pudding, mince-pies, and, for those who desired it, a bottle of beer or mineral waters. The civilian patients also were provided with a similar dinner, all having a good share of turkey and Christmas pudding, the turkeys being the gift of various friends. On Christmas evening, immediately after tea, a concert was given in the soldiers' ward by the nurses, to which as many of the civilian patients as possible were invited. At the close the soldiers cheered sufficiently to raise the roof, and one was heard to remark that he had never had such an enjoyable Christmas Day, and never expected to have such a day in hospital; he had no idea hospitals gave their patients such a good time. On Friday it was the children's tree day, and each child was allowed to invite its mother to tea, the assistant house surgeon, dressed as Father Christmas, distributed the toys off the tree, amidst great excitement from the

**OUR HOSPITALS' CHRISTMAS, 1917.**—(*continued*).

little ones. Through the kindness of Mr. T. Armstrong, a cinematograph performance was given in the ward, all the pictures being such as to amuse children. One little boy wanted to know why a boy and girl were kissing in the pictures, "What's he kissing her for?"

### Crippled Children's Hospital, Alton.

For a week or more before the day at the Crippled Children's Hospital, Alton, the Christmas spirit was moving amongst both patients and staff, and the psychological atmosphere was alive with the magic emotions of the season. The excitement increased as the preparations took on a more or less visible shape, and when the great bunches of holly, laurel, and ivy made their appearance, and enthusiastic sisters and nurses speedily set to work to turn the wards into fairy grottoes, the children were enchanted. Finally came the setting up of the mystic tree with its wonderful fruit, which is the crowning glory of Christmas celebrations to children the whole world over. On Sunday evening before Christmas a party of nurses went in procession round the wards and the estate singing carols. It was an ideal night for the procession; snow lay on the ground, the stars shone brightly, the artificial lights everywhere were turned out, and the scene was a most effective one from the artistic point of view. The distribution of gifts from the Christmas-trees took place on the afternoon of Christmas Day. The patients of each of the four blocks had been gathered for the occasion into one ward on each block, an arrangement which constituted no small part of the fun for the children themselves. The children were first entertained by a party of nurses, who, in dainty costume, went from ward to ward and gave a charming selection of old English songs and dances—just the sort of thing which appeals to children. But the coming of Father Christmas was the event of the day. He came dancing into the wards shouting a boisterous greeting, and went dancing out after keeping everyone in uproarious good spirits.

### Some Christmastide Amusements at Leicester Hospitals.

Continuing the series of entertainments to wounded soldiers, two excellent concerts were given at the Base Hospital on Boxing Day. Lieutenant Irwin's production of "The Rosebuds," a revue written by himself, was presented by the "Happy Returns" party of wounded, and was deservedly and enthusiastically received, and an orchestral concert was given, under the direction of Mr. G. R. Tebbs, by the De Montford Orchestra. Well-known local entertainers added to the evening's amusement. At the invitation of the Commandant, Colonel L. K. Harrison, the Mayor of Leicester, Alderman Jonathan North, J.P., attended to present military medals to Corporal Bevers, R.F.A., and to Lance-Corporal Seeley, of the 2nd East Lancashire Regiment, for exceptional bravery as a stretcher-bearer under heavy fire.

The gracious Christmas messages of the King and Queen were cordially received.

At the North Evington War Hospital a patients' fancy-dress parade was arranged, and selections were given by the R.A.M.C. band. As at the Base Hospital, many competitions were organised, whist drives in the wards, hat-trimming competitions, magic ponds, etc. On Boxing Night a whist drive and entertainment was given in No. 6 ward for Mrs. Alfred Corah by the West-End Association for the Entertainment of Wounded Soldiers, several valuable prizes being competed for. On Thursday evening an entertainment by well-known local artistes was enthusiastically received and much enjoyed. The hospital troupe of Pierrots also largely contributed to the evening's enjoyment.

Lieutenant Irwin's revue, "The Rosebuds," was presented, and enthusiastically welcomed.

At the Royal Infirmary every patient was given at least one useful present on Christmas Day, and each civilian patient was allowed to invite a friend to tea, when a comparison of the Christmas fare showed that institutions this year were able to offer a more liberal dietary than was possible in the homes. As at the military hospitals, there was no lack of entertainment, competitions of various kinds being organised and heartily entered into, and several special teas were provided by friends of the institution during the week. Much amusement was caused in the military wards by the Christmas-trees, each patient being led blindfolded to select the article which fortune held out to him as a claimant. A musical sketch by six of the soldiers was thoroughly enjoyed. The "Song Birds Symphony," presented by Miss Marriott, the assistant matron, and members of the nursing staff, was enthusiastically received, and re-presented during the week by hearty invitation.

At the Groby Road Hospital the wards were tastefully decorated, and concerts arranged by Mr. W. H. Brain, for which Alderman Windley (chairman of the Sanitary Committee of the Town Council) tendered cordial acknowledgment. A successful whist drive for handsome prizes was given, and was followed by an enjoyable dance.

The Groby Road Sanatorium was also decorated. Carol-singing by the St. Martin's Old Boys' Association of thirty voices gave much pleasure to patients and choir, and the latter subsequently gave a sacred concert in the No. 5 ward. At the close Dr. C. K. Millard (M.O.H.) voiced the thanks of the committee and the patients to the Association and their friends for their kind efforts.

At the Knighton V.A.D. Hospital a memorable Christmas was spent, a typical Christmas dinner being provided through the kindness of many good friends. Each patient was the recipient of a Christmas gift, and games were enthusiastically indulged in. Concerts and entertainments were given by various friends.

At the Workhouse and Poor-Law Infirmary special fare was provided, and the Mayor (Alderman North) attended to cheer the inmates and offer the inmates seasonable greetings.

Dinners for the Poor. The seventeenth annual distribution of Christmas dinners for the poor of the town took place on Christmas Day, when 500 dinners, consisting of 2 lb. of roast beef and 2 lb. of potatoes were distributed. Four hundred and seventy-five Christmas parcels of groceries, etc., were also distributed to the blind and the crippled.

For the Blind a distribution of Christmas gifts was made at the Victoria Hall, 160 parcels from the Christmas Dinner Committee and monetary gifts and coal-money by the Wycliffe Society for the Blind. The Leicester Working Men's Club and Institute presented 226 shillings, one for each blind person under the control of the Society and for each blind person in the workhouse. Councillor and Mrs. Wilford presented each blind person with a pair of slippers. On New Year's Day the annual party was held, the hosts being the Morning Star Sundries Co-operative Society. Parcels of groceries, the gift of the Mayor and Mayoress, were presented.

The Christmas of 1917 was altogether a memorable one in the Leicester Institutions, and illustrated the fact that the bonds of sympathy and charity have been unloosed to a degree memorable in the annals of a great city.

## Figures and Tables

**Figure f1:**